# Personalized PET Imaging in Gastric Cancer: An Umbrella Review of Meta‐Analyses to Guide Radiopharmaceutical Selection and Clinical Indication

**DOI:** 10.1155/ijbi/3450212

**Published:** 2026-04-30

**Authors:** Aiganym Amrenova, Alma Shukirbekova, Sholpan Akhelova, Jamilya Assilbayeva, Andrey Gurin, Maira Mukazhanova, Bibigul Kaliyaskarova, Asset Sarsekeyev, Nadiar M. Mussin, Amin Tamadon

**Affiliations:** ^1^ Department of Pharmaceutical Disciplines, Astana Medical University, Astana, Kazakhstan, amu.kz; ^2^ Scientific and Technical Center for Radiochemistry and Isotope Production, Institute of Nuclear Physics, Almaty, Kazakhstan, inp.uz; ^3^ Department of Quality Control of Radiopharmaceuticals, Diagnostic Center, University Medical Center, Astana, Kazakhstan, bvdaihoc.com.vn; ^4^ Radiopharmaceutical Production Department, Center of Nuclear Medicine and Oncology of the Abai Region, Semey, Kazakhstan; ^5^ Department of General Surgery, West Kazakhstan Marat Ospanov Medical University, Aktobe, Kazakhstan; ^6^ Department of Natural Sciences, West Kazakhstan Marat Ospanov Medical University, Aktobe, Kazakhstan

**Keywords:** fibroblast activation protein, fluorodeoxyglucose f18, gastric neoplasms, meta-analysis, positron-emission tomography

## Abstract

**Background:**

Gastric cancer remains a leading cause of cancer‐related morbidity and mortality worldwide, and accurate imaging is critical for diagnosis, staging, recurrence detection, and prognostic assessment. Positron emission tomography (PET), traditionally performed using ^18^F‐fluorodeoxyglucose (FDG), has demonstrated variable performance in gastric cancer, particularly for nodal and peritoneal disease. Recently, ^68^Ga‐labeled fibroblast activation protein inhibitor (FAPI) PET has emerged as a promising alternative, prompting multiple systematic reviews and meta‐analyses with heterogeneous findings.

**Objective:**

We are aimed at synthesizing and appraise meta‐analytic evidence on PET imaging in gastric cancer, with a focus on radiopharmaceutical‐specific performance by clinical indication and certainty of evidence to inform personalized tracer selection.

**Methods:**

An umbrella review was conducted in accordance with PRISMA 2020. The protocol was prospectively registered in the Open Science Framework (OSF). PubMed, Scopus, and Web of Science were searched from inception to December 2025 for systematic reviews and meta‐analyses evaluating PET imaging in gastric cancer. Outcomes included diagnostic accuracy, staging and restaging performance, recurrence detection, prognostic associations, methodological quality (AMSTAR‐2), and certainty of evidence (GRADE).

**Results:**

Eleven meta‐analyses published between 2011 and 2025 were included. FDG PET/CT demonstrated moderate diagnostic performance overall, with high specificity but limited sensitivity for lymph node and peritoneal metastases, while retaining moderate certainty for prognostic assessment based on standardized uptake value (SUV)–derived metrics. In contrast, ^68^Ga‐FAPI PET generally showed higher pooled sensitivity than FDG for primary tumor detection, nodal disease, peritoneal metastases, and recurrence detection across available meta‐analyses, although the certainty of evidence ranged from low to moderate and some findings were derived from mixed‐population reviews.

**Conclusions:**

Available meta‐analytic evidence suggests an indication‐driven, personalized approach to PET imaging in gastric cancer. FDG PET remains useful for prognostic stratification and selected recurrence settings, whereas FAPI PET appears to offer higher diagnostic sensitivity for staging and restaging, particularly for peritoneal disease. Nevertheless, the overall certainty of evidence remains limited by heterogeneity, indirectness, and the absence of updated de novo pooled analyses.

## 1. Introduction

Gastric cancer remains a major global health burden and continues to account for substantial cancer‐related morbidity and mortality despite improvements in prevention, early detection, and multimodality therapy [1]. Contemporary clinical practice guidelines emphasize accurate staging and risk assessment to guide treatment selection and follow‐up, with cross‐sectional imaging as the backbone of evaluation and selective use of functional imaging in specific clinical scenarios [2].

Positron emission tomography (PET), most commonly performed as ^18^F‐fluorodeoxyglucose (FDG) PET/CT, provides whole‐body metabolic assessment and can support detection of distant metastases and evaluation of suspected recurrence [3]. The NCCN Gastric Cancer guidelines (Version 2.2025) and patient guidance acknowledge a role for FDG‐PET/CT in identifying lymph node and distant disease, but do not position it as uniformly mandatory, reflecting recognized limitations and variability in real‐world access and performance [4]. Importantly, FDG uptake in gastric cancer is heterogeneous and can be reduced in diffuse and mucinous/signet‐ring histologies, which contribute to limited sensitivity in some staging settings and create a need for more tailored tracer selection strategies [4].

In parallel with these limitations, there has been rapid clinical expansion of PET radiopharmaceuticals targeting nontumor‐cell compartments [5]. Fibroblast activation protein inhibitor (FAPI) tracers—typically ^68^Ga‐labeled FAPI—target cancer‐associated fibroblasts within the tumor microenvironment and often yield high tumor‐to‐background ratios, particularly in stromal‐rich tumors [6]. Recent evidence syntheses in gastric cancer and broader gastrointestinal malignancies suggest that ^68^Ga‐FAPI PET can outperform FDG‐PET/CT for key indications, notably detection of primary disease, nodal involvement, and peritoneal dissemination, and may improve recurrence evaluation in selected settings [7]. However, the rapidly growing literature has produced multiple systematic reviews and meta‐analyses with varying scope (gastric‐specific vs. mixed cancers), outcomes, and methodological rigor, making it challenging for clinicians and guideline developers to translate this evidence into clear indication‐based recommendations.

To address this gap, an umbrella review of meta‐analyses is timely to consolidate and compare radiopharmaceutical‐specific evidence and to identify where evidence is sufficiently robust to inform clinical choice versus where uncertainty persists. Following modern standards for transparent reporting of evidence syntheses (PRISMA 2020), this umbrella review is aimed at (i) mapping the landscape of meta‐analytic evidence on PET imaging in gastric cancer, (ii) synthesizing tracer‐specific performance by clinical indication (detection, staging, recurrence, and treatment response/prognosis), and (iii) appraising methodological quality and certainty of evidence to guide personalized radiopharmaceutical selection in clinical practice and future research.

## 2. Methods

### 2.1. Protocol Registration

This umbrella review was conducted in accordance with the PRISMA 2020 statement for reporting systematic reviews and meta‐analyses. The review protocol was developed a priori and registered in the Open Science Framework (OSF) (DOI:10.17605/OSF.IO/CXFDG). The protocol defined the objectives, eligibility criteria, outcomes, and evidence synthesis approach prior to study selection and analysis.

### 2.2. Eligibility Criteria

Eligibility criteria were defined using a PICOS framework adapted for imaging‐based evidence synthesis:

Population (*P*): Adults with gastric cancer at any disease stage, including postoperative and recurrent settings. Studies enrolling mixed gastrointestinal or pan‐cancer populations were eligible only if gastric cancer outcomes were reported separately or analyzable as a subgroup.

Index test (*I*): PET‐based imaging, including PET, PET/CT, and PET/MRI, using FDG or ^68^Ga‐labeled FAPI. Reviews including mixed gastrointestinal or pan‐cancer populations were retained only if gastric cancer results were reported separately, gastric cancer formed a prespecified subgroup, or the clinical question directly informed gastric cancer imaging practice; otherwise, such reviews were excluded.

Comparator (*C*): Other imaging modalities, alternative PET tracers, or reference standards such as histopathology and/or clinical‐imaging follow‐up.

Outcomes (*O*): Diagnostic accuracy measures (sensitivity, specificity, likelihood ratios, diagnostic odds ratios [DOR], and area under the curve [AUC]), staging performance (nodal, distant, or peritoneal metastases), recurrence detection, and prognostic outcomes derived from PET parameters.

Study design (*S*): Systematic reviews and meta‐analyses. Narrative reviews, single primary studies, case reports, conference abstracts, and editorials were excluded. For the purposes of this umbrella review, a systematic review was defined as a study that reported a clearly formulated research question, conducted a comprehensive literature search in at least one electronic database, applied explicit eligibility criteria, and performed a structured appraisal and synthesis of included studies. Meta‐analyses were required to include a quantitative synthesis of results. Only articles published in English were considered.

### 2.3. Information Sources and Search Strategy

A comprehensive literature search was performed in PubMed, Scopus, and Web of Science from inception to December 2025. Search strategies combined controlled vocabulary and free‐text terms related to gastric cancer, PET, FDG, FAPI, systematic review, and meta‐analysis (Table 1). The full database‐specific search strategies are presented in Table 1. Reference lists of included reviews were manually screened to identify additional eligible studies.

**Table 1:** Search strategies for PubMed, Scopus, and Web of Science used to identify systematic reviews and meta‐analyses on PET imaging in gastric cancer.

Database	Search field(s)	Search string	Filters/limits
PubMed (MEDLINE)	All fields + MeSH	(((“Stomach Neoplasms”[Mesh]) OR (gastric cancer∗[tiab] OR stomach cancer∗[tiab] OR gastric neoplasm∗[tiab] OR stomach neoplasm∗[tiab] OR gastric carcinoma∗[tiab] OR stomach carcinoma∗[tiab] OR gastric adenocarcinoma∗[tiab])) AND ((“Positron‐Emission Tomography”[Mesh]) OR PET[tiab] OR “positron emission tomograph∗“[tiab]” OR PET/CT[tiab] OR PET‐CT[tiab] OR PET/MRI[tiab] OR PET‐MRI[tiab]) AND ((radiopharmaceutical∗[tiab] OR radiotracer∗[tiab] OR tracer∗[tiab] OR “molecular imaging”[tiab] OR “fluorodeoxyglucose”[tiab] OR FDG[tiab] OR “18F‐FDG”[tiab] OR fluorine‐18[tiab] OR 18F[tiab] OR “11C”[tiab] OR carbon‐11[tiab] OR gallium‐68[tiab] OR 68Ga[tiab] OR zirconium‐89[tiab] OR 89Zr[tiab] OR “FAPI”[tiab] OR “fibroblast activation protein inhibitor”[tiab])) AND ((“Systematic Review”[Publication Type]) OR (meta‐analysis[Publication Type]) OR (systematic review∗[tiab]) OR (meta‐analys∗[tiab]) OR (pooled analys∗[tiab]) OR (umbrella review∗[tiab]) OR (overview of reviews[tiab])))	No date limit initially. Optional: humans. Language: English (only if required by journal).
Scopus	TITLE‐ABS‐KEY	TITLE‐ABS‐KEY ((“gastric cancer∗” OR “stomach cancer∗” OR “gastric neoplasm∗” OR “stomach neoplasm∗” OR “gastric carcinoma∗” OR “stomach carcinoma∗” OR “gastric adenocarcinoma∗”) AND (pet OR “positron emission tomograph∗” OR “pet/ct” OR “pet‐ct” OR “pet/mri” OR “pet‐mri”) AND (radiopharmaceutical∗ OR radiotracer∗ OR tracer∗ OR “molecular imaging” OR fdg OR “18f‐fdg” OR “fluorodeoxyglucose” OR “fluorine‐18” OR “68ga” OR “gallium‐68” OR “89zr” OR “zirconium‐89” OR fapi OR “fibroblast activation protein inhibitor” OR “11c” OR “carbon‐11”) AND (“systematic review” OR “meta‐analysis” OR metaanalys∗ OR “pooled analysis” OR “umbrella review” OR “overview of reviews”))	In Scopus, you can also limit document type to review. Keep “meta‐analysis” terms anyway for precision.
Web of Science Core Collection	TS (topic = title/abstract/keywords)	TS = ((“gastric cancer∗” OR “stomach cancer∗” OR “gastric neoplasm∗” OR “stomach neoplasm∗” OR “gastric carcinoma∗” OR “stomach carcinoma∗” OR “gastric adenocarcinoma∗”) AND (PET OR “positron emission tomograph∗” OR “PET/CT” OR “PET‐CT” OR “PET/MRI” OR “PET‐MRI”) AND (radiopharmaceutical∗ OR radiotracer∗ OR tracer∗ OR “molecular imaging” OR FDG OR “18F‐FDG” OR fluorodeoxyglucose OR “fluorine‐18” OR “68Ga” OR “gallium‐68” OR “89Zr” OR “zirconium‐89” OR FAPI OR “fibroblast activation protein inhibitor” OR “11C” OR “carbon‐11”) AND (“systematic review” OR “meta‐analysis” OR metaanalys∗ OR “pooled analysis” OR “umbrella review” OR “overview of reviews”))	Consider limiting document types = review (WoS filter) after running the search

### 2.4. Study Selection

All records retrieved from the database searches were imported into a reference management software, and duplicate entries were removed prior to screening. Two reviewers independently screened titles and abstracts against the predefined eligibility criteria. Articles that clearly did not meet inclusion criteria were excluded at this stage. Screening was performed in duplicate using EndNote 20 (Clarivate, Philadelphia, Pennsylvania, United States), with disagreements resolved by consensus and third‐reviewer arbitration.

Full texts of potentially eligible articles were then retrieved and assessed independently by the same reviewers. Disagreements regarding eligibility were resolved through discussion and consensus; when necessary, a third reviewer was consulted. Reasons for exclusion at the full‐text stage included absence of a systematic review or meta‐analysis design, insufficient PET‐specific data, lack of quantitative synthesis, or irrelevance to gastric cancer imaging.

### 2.5. Evaluation of Overlap Across Systematic Reviews

Because this study was designed as an umbrella review, overlap of primary studies across included systematic reviews and meta‐analyses was anticipated. To evaluate overlap, we constructed a citation matrix in which rows represented individual primary studies and columns represented the included systematic reviews/meta‐analyses. Presence of a primary study within a given review was coded dichotomously. In addition to visual inspection, overlap was quantified using the corrected covered area (CCA), calculated as follows: CCA = (*N* − *r*)/(rc − *r*), where *N* is the total number of included study occurrences across reviews, *r* is the number of unique primary studies, and *c* is the number of reviews. The degree of overlap was interpreted using conventional thresholds: slight (0%–5%), moderate (6%–10%), high (11%–15%), and very high (> 15%). Because no de novo repooling of primary study data was performed, overlap was interpreted as a measure of redundancy and dependence across reviews rather than as a source of direct statistical double‐counting in a newly generated meta‐analysis.

### 2.6. Data Extraction

Data extraction was performed independently by two reviewers using a predefined and piloted extraction form. For each included systematic review or meta‐analysis, the following information was collected: Bibliographic details (first author, year of publication, and journal), review characteristics (study design, cancer population, number of included primary studies, and total sample size), imaging characteristics (PET modality, radiopharmaceuticals evaluated, and comparator imaging modalities), clinical indications (primary detection, staging, recurrence detection, and prognostic assessment), outcome measures and pooled estimates, measures of heterogeneity (*I* ^2^ or equivalent statistics), risk of bias, or methodological quality assessment tools used by the authors. When data were reported graphically or incompletely, values were extracted from tables or figures where possible. Any discrepancies in extracted data were resolved by consensus.

### 2.7. Risk of Bias of Primary Studies Within Included Reviews

The risk of bias of individual primary studies was not reassessed de novo in this umbrella review. Instead, risk‐of‐bias judgments were extracted as reported in the included systematic reviews and meta‐analyses. These assessments were conducted in the original reviews using established tools appropriate to study design, including QUADAS or QUADAS‐2 for diagnostic accuracy studies and the Newcastle–Ottawa Scale (NOS) or similar instruments for prognostic and observational studies. Reassessment of primary studies was not performed because this overview synthesized evidence at the systematic review/meta‐analysis level, and no reanalysis or repooling of primary study data was undertaken.

### 2.8. Outcomes of Interest

The outcomes of interest were predefined and categorized according to clinical indication and type of evidence. Diagnostic outcomes included the sensitivity and specificity of PET imaging for primary tumor detection, lymph node metastases, peritoneal and distant metastases, and detection of recurrent disease. Where available, secondary diagnostic metrics—such as positive and negative likelihood ratios, DOR, and area under the receiver operating characteristic curve—were also extracted. Staging and restaging performance were assessed based on the accuracy of PET imaging in preoperative and postoperative settings, with particular emphasis on nodal and peritoneal involvement. Prognostic and treatment‐response outcomes focused on associations between PET‐derived quantitative parameters (including standardized uptake value [SUV], SUVmax, and changes in SUV) and survival endpoints, such as overall survival, disease‐free survival, progression‐free survival, and relapse‐free survival. In addition, the certainty of evidence across outcomes was evaluated using the GRADE framework, adapted for diagnostic test accuracy and prognostic evidence, and summarized across radiopharmaceuticals and clinical indications. Collectively, these outcomes formed the basis for the qualitative synthesis, comparative interpretation of radiopharmaceutical performance, and certainty‐of‐evidence assessment presented in the Results section.

### 2.9. Methodological Quality Assessment

The methodological quality of the included systematic reviews and meta‐analyses was assessed using the AMSTAR‐2 tool. Two reviewers independently evaluated each review across critical and noncritical domains, including protocol registration, literature search, study selection, data extraction, risk‐of‐bias assessment, and consideration of publication bias. Any discrepancies were resolved by consensus. Overall confidence in the results of each review was categorized as high, moderate, low, or critically low, in accordance with AMSTAR‐2 guidance. The domain‐level assessments and overall confidence ratings were synthesized and visualized to inform interpretation of the evidence base.

Assessment of reporting bias was based on information provided in the included systematic reviews and meta‐analyses. Where available, results of publication bias assessments—such as funnel plot inspection, Egger′s regression test, Begg′s test, or related methods—were extracted as reported. When publication bias was not assessed or not reported in the original reviews, this was recorded as “not reported.” No additional statistical tests for reporting bias were conducted in this umbrella review, as no reanalysis or repooling of primary study data was undertaken.

### 2.10. Evidence Synthesis Approach

A narrative and comparative evidence synthesis was undertaken, consistent with the umbrella‐review methodology. Findings were synthesized at the meta‐analysis level rather than repooling primary studies. Diagnostic and prognostic outcomes were summarized by radiopharmaceutical and clinical indication, with pooled estimates extracted directly from the included meta‐analyses. Where multiple meta‐analyses addressed the same tracer–indication pair, results were compared qualitatively, and summary interpretations emphasized direction and magnitude of effects, consistency, and clinical relevance, rather than generating new pooled estimates. Visual summaries were used to support interpretation, including quantitative comparison plots and indication‐based decision frameworks. Between‐review heterogeneity was explored qualitatively by comparing reported heterogeneity statistics, clinical contexts (population characteristics, disease stage, and clinical indication), imaging protocols, reference standards, and outcome definitions across meta‐analyses. No new subgroup analyses or meta‐regressions were performed, as this umbrella review did not repool primary study data and synthesized evidence at the meta‐analysis level. No de novo quantitative reanalysis or sensitivity analysis of primary studies was performed because this study was designed as an umbrella review synthesizing evidence at the level of published systematic reviews and meta‐analyses. Accordingly, our findings reflect comparative appraisal of existing pooled evidence rather than generation of new pooled estimates. During synthesis, evidence from gastric cancer–specific reviews was prioritized, whereas findings from mixed‐population reviews were treated as indirect supportive evidence and interpreted with greater caution.

### 2.11. Certainty of Evidence

The certainty of evidence across outcomes was assessed using the GRADE framework, adapted for diagnostic test accuracy and prognostic evidence. For diagnostic outcomes, certainty judgments considered risk of bias, inconsistency, indirectness, imprecision, and publication bias, with starting certainty based on the body of evidence summarized by each meta‐analysis and subsequent downgrading as appropriate. For prognostic outcomes, standard GRADE considerations for observational evidence were applied, including assessment of heterogeneity, precision of effect estimates, and potential confounding. Certainty ratings were summarized by outcome, radiopharmaceutical, and clinical indication, and presented in structured tables and visual heatmaps to facilitate transparent interpretation.

### 2.12. Software and Codes

All stages of study management, data extraction, synthesis, and visualization were conducted using a combination of statistical software, reference management tools, and custom‐written scripts to ensure transparency and reproducibility. Bibliographic records were managed and deduplicated using standard reference management software prior to screening.

Data handling, tabulation, and generation of figures were primarily performed in R (Version 4.5.2) using RStudio (Version 2025.09.1 Build 401; Posit Software, Boston, Massachusetts, United States) as the integrated development environment. Custom R scripts were developed for data cleaning, transformation, and visualization, including generation of heatmaps, comparative plots, and traffic‐light matrices summarizing radiopharmaceutical performance and certainty of evidence. Core R packages used included tools for data manipulation and graphics, as well as packages for reading spreadsheet files and managing structured datasets.

Methodological quality assessments (AMSTAR‐2) and certainty‐of‐evidence evaluations (GRADE) were implemented using predefined decision rules operationalized within spreadsheets and verified using R‐based visual summaries. No reanalysis or repooling of primary study data was undertaken; all quantitative values were extracted directly from published meta‐analyses.

## 3. Results

### 3.1. Study Selection

**Figure 1:**
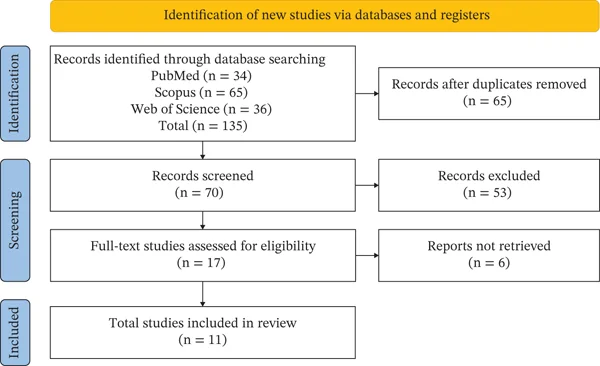
Flow diagram illustrating identification, screening, eligibility assessment, and inclusion of systematic reviews and meta‐analyses on PET imaging in gastric cancer.

A total of 135 records were identified through database searching (PubMed *n* = 34, Scopus *n* = 65, and Web of Science *n* = 36). After removal of duplicates, 65 unique records remained for title and abstract screening. Of these, 53 records were excluded based on the predefined eligibility criteria. Seventeen reports were sought for full‐text retrieval and eligibility assessment. Six reports could not be retrieved. The remaining 11 systematic reviews and meta‐analyses were included in the umbrella review (Figure 1).

### 3.2. Characteristics of Included Meta‐Analyses

A total of 11 systematic reviews and meta‐analyses, published between 2011 and 2025, were included (Tables 2 and 3). These reviews evaluated the clinical utility of PET imaging in gastric cancer across diagnostic, staging, recurrence detection, and prognostic settings, encompassing both established and emerging radiopharmaceuticals.

**Table 2:** Characteristics of included systematic reviews and meta‐analyses evaluating PET radiopharmaceuticals in gastric cancer.

Author, year (reference)	Journal	Review type	Cancer type(s)	Population specificity	Radiopharmaceutical(s)	Comparator	Clinical indication	No. of included studies	Total patients
Choi et al., 2023 [10]	Yeungnam Medical Science	Systematic review and meta‐analysis (diagnostic accuracy)	Gastric cancer (postcurative resection recurrence)	Gastric cancer only	^18^F‐FDG PET or PET/CT	Not pooled as head‐to‐head; evaluates PET/PET‐CT diagnostic performance versus reference standards in included studies	Detection of recurrent gastric cancer after curative resection	17	1732
Kaneko et al., 2015 [11]	Journal of Nuclear Medicine	Systematic review (FDG avidity predictors) + retrospective cohort + PET scoring system	Gastric adenocarcinoma	Gastric cancer only	^18^F‐FDG PET/PET‐CT	Not a head‐to‐head comparator; identifies predictors of FDG avidity (and selection tool)	Patient selection for staging FDG PET (improving yield)	Systematic review: 18 articles; retrospective cohort: 40 patients	Cohort: 40; SR patient totals not aggregated as a single number in excerpt
Ruan et al., 2023 [12]	Theranostics	Systematic review amd meta‐analysis (FAPI PET)	Gastric cancer (incl. primary, recurrence, LN, distant/peritoneal mets)	Gastric cancer only	^68^Ga‐FAPI (mostly FAPI‐04; some studies include other FAPI compounds)	^18^F‐FDG PET/CT (comparative evidence)	Early diagnosis, initial staging, detection of recurrence/metastases; tracer selection (FAPI vs. FDG)	14	527 total (358 GC)
Seevaratnam et al., 2012 [13]	Gastric Cancer (Suppl 1)	Systematic review and meta‐analysis of preoperative imaging accuracy	Gastric cancer (newly diagnosed; preoperative staging)	Gastric cancer only	^18^F‐FDG PET (as one modality)	AUS, CT, MRI versus pathology	Preoperative TNM staging (accuracy vs. pathology)	40	3758
Wang et al., 2023 [14]	European Journal of Medical Research	Meta‐analysis (comparative trials only)	Gastric cancer	Gastric cancer only	[^68^Ga]Ga‐FAPI‐04 PET MRI/CT	[^18^F]‐FDG PET MRI/CT	Detection of primary tumor; LN metastases; peritoneal involvement	5	148
Wang and Chen, 2011 [15]	BMC Gastroenterology	Systematic review (diagnostic test accuracy) + meta‐analysis	Gastric cancer (hepatic and peritoneal metastases)	Gastric cancer only	^18^F‐FDG PET	US, EUS, CT, and MRI	Detecting liver and peritoneal metastases pretherapeutically	33	Total patients not stated in the extracted excerpt (study sizes varied by modality)
Wu et al., 2012 [8]	BMC Cancer	Meta‐analysis (prognostic)	Gastric cancer	Gastric cancer only	^18^F‐FDG PET (SUV)	High SUV versus low SUV groups (cutoffs varied)	Prognosis prediction (pretreatment PET SUV)	8	1080
Wu et al., 2024 [16]	Translational Cancer Research	Systematic review and meta‐analysis (diagnostic recurrence)	All cancers (subgroup: GI/incl. gastric)	Pan‐cancer recurrence review with GI/gastric subgroup	[^68^Ga]Ga‐FAPI‐04 PET	[^18^F]FDG PET	Detection of cancer recurrence (incl. GI recurrence subgroup)	12	224
Zhang et al., 2021 [17]	Experimental and Therapeutic Medicine	Systematic review and meta‐analysis (diagnostic accuracy)	Gastric cancer (primary staging + recurrence)	Gastric cancer only	^18^F‐FDG PET/CT	CECT + histopathology reference standard	Primary TNM staging (N/M) + diagnosis of recurrent gastric cancer	58	9997
Zhu et al., 2012 [9]	British Journal of Radiology	Systematic review and meta‐analysis (prognostic)	Esophagogastric junction adenocarcinoma (localized; includes gastric cardia/AEG)	Esophagogastric junction/upper GI related	^18^F‐FDG PET (SUVmax, *Δ*SUV)	Metabolic responders versus nonresponders; different PET time‐points (PET1 vs. PET2/PET3)	Prognostic stratification and early response assessment during neoadjuvant therapy	10 (meta‐analysis evaluable studies)	798
Zou and Zhao, 2013 [18]	Surgical Oncology	Systematic review and meta‐analysis (diagnostic accuracy)	Gastric cancer (postsurgical patients)	Gastric cancer only	^18^F‐FDG PET/CT	Reference standard: histopathology and/or clinical and imaging follow‐up; compared indirectly with CT/endoscopy in included studies	Detection of gastric cancer recurrence after curative surgical resection	8	500

Abbreviations: ^18^F‐FDG, fluorine‐18 fluorodeoxyglucose; ^68^Ga, gallium‐68; *Δ*SUV, change in standardized uptake value; AEG, adenocarcinoma of the esophagogastric junction; CT, computed tomography; CECT, contrast‐enhanced computed tomography; DFS, disease‐free survival; EUS, endoscopic ultrasonography; FAPI, fibroblast activation protein inhibitor; FAPI‐04, fibroblast activation protein inhibitor‐04; FDG, ^18^F‐fluorodeoxyglucose; GI, gastrointestinal; GC, gastric cancer; LN, lymph node; MRI, magnetic resonance imaging; OS, overall survival; PET, positron emission tomography; PET/CT, positron emission tomography/computed tomography; PET/MRI, positron emission tomography/magnetic resonance imaging; SR, systematic review; SUV, standardized uptake value; SUVmax, maximum standardized uptake value; US, ultrasonography; TNM, tumor–node–metastasis.

**Table 3:** Summary of outcomes and methodological quality of included systematic reviews and meta‐analyses evaluating PET radiopharmaceuticals in gastric cancer.

Author, year (reference)	Outcomes reported	Main findings	Heterogeneity (*I* ^2^)	Risk‐of‐bias tool
Choi et al., 2023 [10]	Sens/spec, LR+/LR−, DOR, AUC	PET/PET‐CT showed good sensitivity (0.82) and specificity (0.86) for recurrence detection	Sensitivity *I* ^2^ = 76.5*%*; specificity *I* ^2^ = 94.2*%*	Modified QUADAS‐2
Kaneko et al., 2015 [11]	Detection rate/FDG avidity; predictors; scoring system performance (sens/spec)	Predictors: larger size, non‐SRC histology, GLUT1+; scoring system sensitivity 85% and specificity 71%	*I* ^2^ not reported	Not reported (focus is predictor synthesis + model development)
Ruan et al., 2023 [12]	Pooled sensitivity/specificity and AUC; subgroup sensitivities by lesion site; SUVmax & TBR comparisons	^68^Ga‐FAPI shows higher pooled sensitivity and AUC than FDG; superior sensitivity for primary, recurrence, LN, distant and peritoneal mets	*I* ^2^ not reported as a single overall for diagnostic accuracy; heterogeneity reported for quantitative pooling	QUADAS‐2
Seevaratnam et al., 2012 [13]	Accuracy, overstaging/understaging, kappa, sensitivity, specificity (by modality)	PET: lowest sensitivity but highest specificity for *N* staging; no modality clearly superior for *M* staging	*I* ^2^ not presented (pooling reported as pooled means ± SE and comparisons)	Not specified as a single named tool in the excerpt (methods focus on recalculating diagnostic performance, pooling approach)
Wang et al., 2023 [14]	Sensitivity (patient‐based; LN; peritoneal)	FAPI‐04 PET MRI/CT superior to FDG for primary, LN, peritoneal detection	(From figures) substantial heterogeneity reported for pooled sensitivity in patient‐based outcomes (example shown *I* ^2^ = 84*%* in patient‐based analysis)	CASP checklist
Wang and Chen, 2011 [15]	Pooled sensitivity/specificity/DOR (by modality, liver vs. peritoneum)	CT most sensitive for liver mets; EUS highest sensitivity for peritoneal mets but low; modalities not consistently high sensitivity/specificity	*I* ^2^ not reported; heterogeneity assessed via Cochrane′s *Q* and threshold effect tests	QUADAS
Wu et al., 2012 [8]	HR for OS and RFS	High pretreatment SUV associated with worse OS and RFS	*I* ^2^ = 0*%* (OS) and *I* ^2^ = 0*%* (RFS)	Newcastle–Ottawa Scale (NOS)
Wu et al., 2024 [16]	Pooled sensitivity/specificity; GI recurrence subgroup	FAPI higher sensitivity than FDG for recurrence; GI subgroup: FAPI sensitivity 1.00 versus FDG 0.57; specificity similar/low precision	Uses *I* ^2^ to assess heterogeneity, but *I* ^2^ values not provided in the extracted abstract snippet	QUADAS‐2
Zhang et al., 2021 [17]	Sens/spec (plus DOR, LR+/LR−, AUC) for *N*, *M*, recurrence	PET/CT and CECT useful overall; PET/CT high specificity; limited for ruling in/out LN or recurrence; both useful for confirming distant metastasis	N staging (PET): sens *I* ^2^ = 87.6*%*, spec *I* ^2^ = 64.2*%*; M staging (PET): sens *I* ^2^ = 83.5*%*, spec *I* ^2^ = 94.1*%*; Recurrence (PET): sens *I* ^2^ = 75.7*%*, spec *I* ^2^ = 89.7	QUADAS‐2
Zhu et al., 2012 [9]	Hazard ratios for OS and DFS based on SUVmax and changes in SUV	Relative changes in SUVmax (PET1 → PET2/PET3) strongly predicted OS and DFS; baseline SUVmax alone had limited prognostic value; supports personalized treatment adaptation	OS (*Δ*SUV): *I* ^2^ = 16*%*; DFS (*Δ*SUV): *I* ^2^ = 18*%*; several analyses showed low heterogeneity	Custom methodological quality scoring system (modified PET‐specific scale)
Zou and Zhao, 2013 [18]	Sensitivity, specificity, PLR, NLR, DOR, and HSROC AUC	^18^F‐FDG PET/CT demonstrated moderate diagnostic accuracy for detecting recurrence (pooled sensitivity 0.86; specificity 0.88; AUC 0.93); useful for confirming recurrence but insufficient alone to rule it out	Sensitivity *I* ^2^ ≈ 83.7%; specificity *I* ^2^ ≈ 75.8%	QUADAS

*Note:* Reviews including mixed gastrointestinal or pan‐cancer populations were considered indirect evidence for gastric cancer and were weighted cautiously during narrative synthesis and GRADE assessment.

Abbreviations: *Δ*SUV, change in standardized uptake value; AUC, area under the curve; CASP, critical appraisal skills program; DM, distant metastasis; DOR, diagnostic odds ratio; DFS, disease‐free survival; FAPI, fibroblast activation protein inhibitor; FDG, ^18^F‐fluorodeoxyglucose; GI, gastrointestinal; HR, hazard ratio; HSROC, hierarchical summary receiver operating characteristic; *I* ^2^, Higgins′ inconsistency index; JBI, Joanna Briggs Institute; LN, lymph node; LNM, lymph node metastasis; LR−, negative likelihood ratio; LR+, positive likelihood ratio; NOS, Newcastle–Ottawa Scale; OS, overall survival; PET, positron emission tomography; PFS, progression‐free survival; QUADAS, quality assessment of diagnostic accuracy studies; RFS, relapse‐free survival; Sens, sensitivity; Spec, specificity; SUV, standardized uptake value; SUVmax, maximum standardized uptake value; TBR, tumor‐to‐background ratio.

Of the 11 included meta‐analyses, 8 (72.7%) evaluated diagnostic accuracy outcomes, 3 (27.3%) included prognostic analyses, and 2 (18.2%) assessed treatment response. Five meta‐analyses evaluated lymph node metastases, four assessed peritoneal metastases, and three evaluated recurrence detection. Two reviews specifically examined PET‐based metrics for early treatment response and prognosis using SUV‐derived indices.

The majority of evidence concerned ^18^F‐FDG PET or PET/CT, reflecting its long‐standing role in gastric cancer imaging. More recent reviews incorporated ^68^Ga‐FAPI PET, either as a direct comparator to FDG or within mixed‐modality analyses. Comparators across studies included contrast‐enhanced CT, MRI, ultrasonography, endoscopic ultrasonography, and reference standards such as histopathology and clinical or imaging follow‐up.

The number of primary studies per meta‐analysis ranged from 5 to 58, with total patient numbers varying from 148 to nearly 10,000, depending on the clinical question and publication period. Diagnostic accuracy meta‐analyses generally pooled larger populations, whereas comparative FAPI‐focused analyses and prognostic reviews involved fewer studies and patients. Although most reviews focused exclusively on gastric cancer, some recurrence‐ or prognosis‐oriented analyses included mixed gastrointestinal or pan‐cancer cohorts with gastric cancer evaluated as a predefined subgroup. Accordingly, the disease‐specific strength of evidence was considered highest for gastric cancer–only meta‐analyses and lower for mixed gastrointestinal or pan‐cancer reviews, which were interpreted as indirect evidence.

Reported outcomes included sensitivity, specificity, likelihood ratios, DOR, and AUC curve for diagnostic performance, and hazard ratios for overall, disease‐free, progression‐free, or relapse‐free survival for prognostic analyses. Overall, FDG PET/CT demonstrated moderate‐to‐good diagnostic performance, often with higher specificity than sensitivity. Comparative meta‐analyses consistently indicated higher sensitivity and lesion detectability with ^68^Ga‐FAPI PET, particularly for primary tumors, nodal disease, and peritoneal metastases. High pretreatment SUV was significantly associated with poorer overall survival (HR > 1.5) and recurrence‐free survival, with no heterogeneity (*I* ^2^ = 0*%*) in pooled analyses [8]. Changes in SUV during treatment (*Δ*SUV) showed stronger prognostic value for both OS and DFS compared with baseline SUV, with low heterogeneity (*I* ^2^ = 16*%*–18*%*) [9].

Substantial between‐study heterogeneity was common in diagnostic accuracy analyses (*I* ^2^ frequently > 70*%*), reflecting differences in patient populations, imaging protocols, and reference standards. In contrast, prognostic meta‐analyses based on SUV changes generally showed low to moderate heterogeneity. Methodological quality was assessed using established tools—most commonly QUADAS/QUADAS‐2 for diagnostic reviews and NOS, CASP, or JBI checklists for prognostic analyses. Overall, the included meta‐analyses were of moderate methodological quality, supporting synthesis while underscoring ongoing variability in evidence consistency.

### 3.3. Radiopharmaceutical‐Specific Evidence Synthesis

Radiopharmaceutical‐specific evidence across the included meta‐analyses demonstrates substantial heterogeneity, with reported *I* ^2^ values ranging from 64.2% to 94.2% for diagnostic specificity and sensitivity across meta‐analyses (Table 3).

#### 3.3.1. ^18^F‐FDG PET

Evidence synthesized from diagnostic accuracy meta‐analyses indicates that ^18^F‐FDG PET/CT shows high specificity but limited sensitivity, particularly for early‐stage disease, lymph node involvement, and peritoneal metastases. In preoperative staging, FDG‐PET exhibited the lowest sensitivity for nodal staging but the highest specificity among imaging modalities, limiting its value as a standalone staging tool while supporting its role in confirming advanced disease [15]. Similarly, in the assessment of hepatic and peritoneal metastases, FDG‐PET did not demonstrate consistently high sensitivity, especially for peritoneal spread, where false‐negative results were frequent [14].

**Figure 2:**
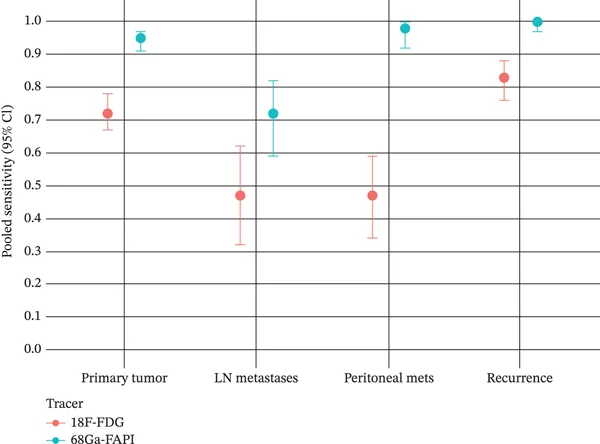
Summarizes the radiopharmaceutical‐specific diagnostic performance derived from pooled estimates across meta‐analyses, highlighting clear and consistent differences between tracers across clinical indications. In particular, the magnitude of separation between FDG and FAPI pooled sensitivities increases progressively from primary tumor detection to lymph node and peritoneal metastases, underscoring the indication‐dependent value of tracer selection.

In contrast, FDG‐PET provided robust prognostic information. Meta‐analytic evidence shows that high pretreatment SUV values are significantly associated with worse overall survival and recurrence‐free survival, supporting FDG‐PET as a tool for metabolic risk stratification rather than purely for anatomical staging [16]. These findings are visually reflected in Figure 2, where FDG‐PET demonstrates modest pooled sensitivity for lymph node and peritoneal metastases compared with primary tumor detection and recurrence, reinforcing its limited role in detecting low‐volume or infiltrative disease. For recurrence detection, pooled sensitivity and specificity reached 0.82 and 0.86, respectively [10], with high heterogeneity (*I* ^2^ = 76.5*%*and 94.2*%*). For nodal staging, sensitivity was as low as 0.40–0.60 across studies, whereas specificity exceeded 0.85.

#### 3.3.2. ^68^Ga‐FAPI PET

More recent meta‐analyses consistently demonstrate the superiority of ^68^Ga‐FAPI PET over FDG‐PET for diagnostic indications in gastric cancer. FAPI‐PET showed higher pooled sensitivity and AUC for detection of primary tumors, local recurrence, lymph node metastases, distant metastases, and especially peritoneal metastases, where FDG‐PET performance is poorest [12]. Quantitative analyses further revealed higher SUVmax and tumor‐to‐background ratios with FAPI‐PET, reflecting low physiological uptake in normal gastric and peritoneal tissues.

Comparative meta‐analyses confirmed that [^68^Ga]Ga‐FAPI‐04 PET MRI/CT outperforms FDG‐PET MRI/CT in detecting primary tumors, nodal disease, and peritoneal involvement, with clinically meaningful gains in sensitivity across patient‐based analyses [8]. These findings position FAPI‐PET as a promising tracer for initial staging, detection of recurrence, and evaluation of metastatic spread, particularly in histological subtypes with low FDG avidity. As illustrated in Figure 2, FAPI‐PET consistently achieves near‐ceiling pooled sensitivity for peritoneal metastases and recurrence, with narrower confidence intervals than FDG‐PET, suggesting both improved detection and greater robustness across studies. Meta‐analysis by Ruan et al. [12] reported pooled sensitivity exceeding 0.90 for primary tumor and peritoneal metastases, with higher AUC compared with FDG. In comparative analysis, FAPI sensitivity for GI recurrence reached 1.00 versus 0.57 for FDG [16].

#### 3.3.3. Tracer Selection by Clinical Indication

Taken together, the evidence supports a personalized radiopharmaceutical selection strategy. FDG‐PET retains value in prognostic stratification and in confirming metabolically active advanced disease, whereas FAPI‐PET provides superior diagnostic sensitivity for staging and restaging, especially for peritoneal and nodal disease. Dual‐tracer approaches, although supported only by limited evidence, may improve diagnostic confidence in selected cases; however, this is currently supported by limited evidence from small comparative studies and requires further validation [12]. Accordingly, Figure 2 provides a pragmatic visual framework for tracer selection in clinical practice, favoring FDG‐PET for prognostic assessment and metabolically active recurrence, and FAPI‐PET for comprehensive staging and restaging, particularly in patients at risk for peritoneal or nodal dissemination.

#### 3.3.4. Summary Framework for Radiopharmaceutical Selection

To translate the heterogeneous meta‐analytic evidence into clinically actionable guidance, Table 4 summarizes radiopharmaceutical‐specific performance across major clinical indications in gastric cancer. The table integrates pooled diagnostic and prognostic findings from multiple meta‐analyses, emphasizing relative strengths and limitations of each tracer rather than absolute performance metrics.

**Table 4:** Radiopharmaceutical‐specific evidence summary by clinical indication in gastric cancer.

Radiopharmaceutical	Primary staging	LN metastases	Peritoneal metastases	Recurrence detection	Prognostic value
18F‐FDG	Moderate diagnostic performance; limited sensitivity in early or diffuse tumors	Low sensitivity, high specificity; useful for confirming advanced nodal disease	Low sensitivity; frequent false‐negative results	Moderate; acceptable sensitivity for clinically suspected recurrence	High; SUV‐based metrics consistently associated with survival outcomes
^68^Ga‐FAPI	High sensitivity for primary tumor detection	High sensitivity for nodal involvement	Very high sensitivity; superior detection of peritoneal dissemination	High; near‐ceiling sensitivity for recurrence	Emerging; prognostic utility not yet established in meta‐analyses

Abbreviations: ^18^F, fluorine‐18; ^68^Ga, gallium‐68; FAPI, fibroblast activation protein inhibitor; FDG, ^18^F‐fluorodeoxyglucose; LN, lymph node; PET, positron emission tomography; SUV, standardized uptake value.

As shown in Table 4, ^18^F‐FDG PET demonstrates moderate diagnostic performance for primary staging and recurrence detection, with consistently limited sensitivity for lymph node and peritoneal metastases, but retains strong prognostic value based on SUV‐derived parameters. In contrast, ^68^Ga‐FAPI PET exhibits high to very high diagnostic sensitivity across staging and restaging indications, particularly for peritoneal dissemination, whereas its prognostic utility remains insufficiently established due to the current absence of dedicated survival meta‐analyses.

### 3.4. Indication‐Based Performance

#### 3.4.1. Detection of Primary Disease

Across included meta‐analyses, PET tracer performance for primary tumor detection varies substantially by radiopharmaceutical and tumor biology. ^18^F‐FDG PET demonstrates moderate sensitivity for detecting primary gastric tumors, with reduced performance in early‐stage disease and histological subtypes characterized by low glucose metabolism, such as signet‐ring cell and diffuse‐type carcinomas. In contrast, ^68^Ga‐FAPI PET consistently shows high sensitivity for primary lesion detection, attributable to its targeting of cancer‐associated fibroblasts and low physiological uptake in the normal gastric wall. These features enable improved lesion conspicuity and more reliable detection of infiltrative or stromal‐rich tumors.

#### 3.4.2. Staging (Nodal and Distant Disease)

For staging purposes, particularly lymph node and peritoneal involvement, tracer‐dependent differences are pronounced. Meta‐analytic evidence indicates that ^18^F‐FDG PET has limited sensitivity for nodal metastases, despite relatively high specificity, reflecting its inability to detect small‐volume or low‐metabolic nodal disease. Performance is even poorer for peritoneal metastases, where physiological bowel uptake and low tumor glycolytic activity contribute to frequent false‐negative findings.

**Figure 3:**
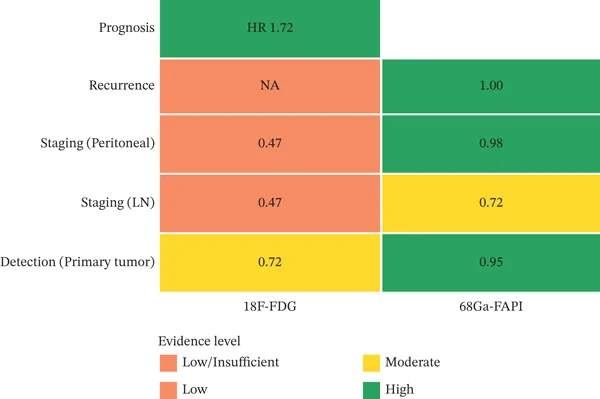
Indication‐based summary of PET radiopharmaceutical performance in gastric cancer. Diagnostic indications are classified according to pooled sensitivity estimates from meta‐analyses, whereas prognostic performance reflects pooled hazard ratios for overall survival where available. Color coding indicates relative evidence level (green, high; yellow, moderate; red, low/insufficient), and numeric values represent pooled point estimates.

In contrast, ^68^Ga‐FAPI PET demonstrates high to very high sensitivity for both lymph node and peritoneal metastases, with the greatest relative advantage observed in peritoneal dissemination. These findings support the preferential use of FAPI‐based imaging for comprehensive staging, particularly when accurate assessment of operability and metastatic burden is clinically critical. As summarized in Figure 3, FAPI‐PET demonstrates consistently high performance for primary tumor detection, whereas FDG‐PET achieves only moderate sensitivity.

#### 3.4.3. Detection of Recurrence

The detection of tumor recurrence represents a key clinical indication for PET imaging following curative treatment. Meta‐analyses consistently show that ^18^F‐FDG PET provides moderate sensitivity for recurrence detection, particularly in metabolically active disease, but may miss low‐volume, fibrotic, or infiltrative recurrent lesions.

By contrast, ^68^Ga‐FAPI PET achieves near‐ceiling sensitivity for recurrence detection, including gastrointestinal recurrence subgroups, outperforming FDG‐PET across pooled analyses. These results suggest a clear role for FAPI‐PET in restaging and surveillance settings, especially when conventional imaging or FDG‐PET yields equivocal findings.

#### 3.4.4. Treatment Response and Prognostic Assessment

Evidence for treatment response assessment and prognostic stratification is currently strongest for ^18^F‐FDG PET. Meta‐analyses demonstrate that elevated pretreatment SUV values are significantly associated with worse overall and recurrence‐free survival, supporting FDG‐derived metabolic parameters as validated prognostic biomarkers.

In contrast, although ^68^Ga‐FAPI PET shows superior diagnostic performance, robust meta‐analytic evidence supporting its role in prognostic stratification or treatment response monitoring remains limited. Consequently, FAPI‐PET should currently be regarded as an emerging tool in this domain, pending dedicated longitudinal and survival‐focused studies.

Importantly, the indication‐based framework shown in Figure 3 should be interpreted as a decision‐support tool rather than a prescriptive guideline. The observed performance gradients reflect current evidence derived from secondary synthesis and may evolve as additional prospective and prognostic studies—particularly for FAPI‐based imaging—become available.

### 3.5. Methodological Quality and Risk of Bias Across Studies

**Figure 4:**
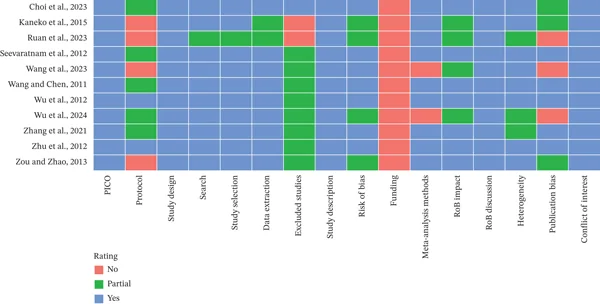
Heatmap of AMSTAR‐2 domain‐level ratings for the included systematic reviews and meta‐analyses on PET imaging in gastric cancer.

The methodological quality of the included systematic reviews and meta‐analyses was assessed using the AMSTAR‐2 tool, and the domain‐level ratings are summarized in Figure 4. Overall, the quality of the evidence base was heterogeneous, with studies demonstrating variable compliance across critical and noncritical AMSTAR‐2 domains.

Most reviews adequately addressed the PICO framework, study design, literature search, study selection, and data extraction, indicating generally sound methodological foundations. In contrast, protocol registration was inconsistently reported, representing one of the most frequently unmet domains. In several studies, the absence of a registered or clearly accessible protocol limited transparency regarding a priori methodological decisions.

Assessment of excluded studies and provision of sufficient study descriptions were commonly reported; however, explicit justification for exclusions was often incomplete. Domains related to funding sources of primary studies were poorly reported across the majority of reviews, constituting a recurrent methodological weakness.

Regarding synthesis and interpretation, most meta‐analyses appropriately described meta‐analytic methods, addressed the impact of risk of bias on the results, and discussed between‐study heterogeneity. Nevertheless, formal evaluation of publication bias was absent or only partially addressed in several reviews, particularly those including a limited number of primary studies.

### 3.6. Certainty of Evidence Across Outcomes

**Figure 5:**
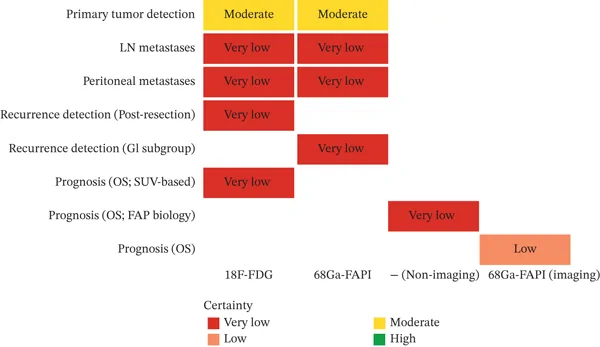
Certainty of evidence (GRADE) for diagnostic and prognostic performance of ^18^F‐FDG and ^68^Ga‐FAPI PET across clinical outcomes in gastric cancer.

The certainty of evidence across diagnostic and prognostic outcomes was assessed using the GRADE framework, with results summarized in Figure 5. Overall, the certainty of evidence varied substantially by clinical indication and radiopharmaceutical, reflecting differences in study design, population heterogeneity, and availability of outcome‐specific meta‐analyses.

For primary tumor detection, the certainty of evidence was rated as moderate for both ^18^F‐FDG and ^68^Ga‐FAPI PET, supported by pooled sensitivity estimates with relatively narrow confidence intervals. However, the evidence was downgraded due to risk of bias in patient selection and incomplete reporting of heterogeneity across diagnostic subgroups.

In contrast, for lymph node and peritoneal metastases, the certainty of evidence was very low for both tracers. This downgrading was primarily driven by substantial heterogeneity, methodological limitations, and imprecision, particularly related to small lesion size, variable reference standards, and inconsistent reporting across studies.

For recurrence detection, the certainty of evidence remained very low. Although pooled sensitivity estimates were favorable—especially for ^68^Ga‐FAPI PET—the evidence base was limited by retrospective study designs, small sample sizes, and mixed gastrointestinal populations, leading to downgrading for risk of bias, indirectness, and imprecision.

Regarding prognostic outcomes, ^18^F‐FDG PET demonstrated moderate certainty based on consistent associations between SUV‐derived parameters and overall survival. Nonetheless, the observational nature of the included cohorts and variability in SUV cut‐off definitions warranted downgrading for risk of bias. For FAPI‐based imaging, no dedicated prognostic meta‐analysis was available, resulting in a very low certainty of evidence for survival outcomes.

### 3.7. Overlap of Primary Studies

**Figure 6:**
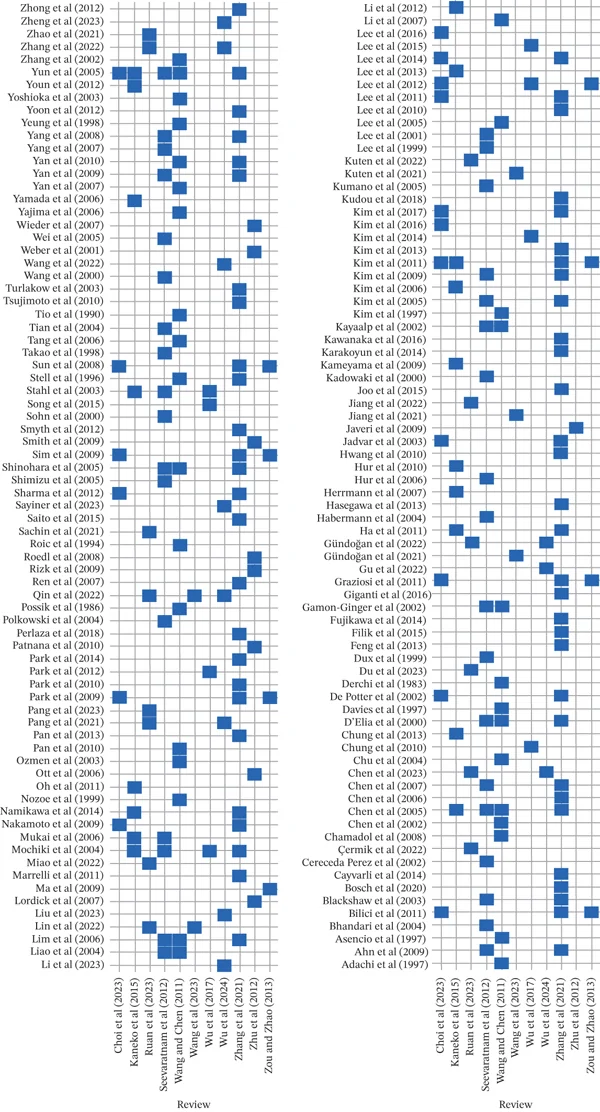
Citation matrix showing overlap of primary studies across included systematic reviews and meta‐analyses. Rows represent unique primary studies and columns represent included reviews. Shaded cells indicate inclusion of a primary study within a given review. Quantitative overlap was additionally assessed using corrected covered area (CCA).

Figure 6 presents the citation matrix illustrating overlap of primary studies across the included systematic reviews and meta‐analyses. Quantitative overlap assessment showed a CCA of 4.08%, indicating slight overlap among the included reviews. Visual inspection further confirmed partial but not complete overlap, with several primary studies appearing in multiple reviews, particularly among FDG PET–focused meta‐analyses addressing similar clinical indications such as recurrence detection and preoperative staging.

Overlap was most pronounced among earlier FDG PET meta‐analyses, reflecting reliance on a shared set of foundational studies. In contrast, more recent meta‐analyses—especially those evaluating ^68^Ga‐FAPI PET—demonstrated limited overlap, consistent with the emerging nature of this evidence base and the inclusion of newer primary studies not captured in earlier reviews.

Importantly, no single systematic review dominated the evidence base, and multiple reviews contributed unique primary studies, supporting the inclusion of all eligible meta‐analyses despite some degree of redundancy. Given that evidence synthesis was conducted at the review level and no de novo pooling of primary study data was performed, the observed overlap was not expected to introduce substantial bias into the conclusions of this umbrella review.

## 4. Discussion

This umbrella review synthesizes meta‐analytic evidence on personalized PET imaging in gastric cancer, focusing on how radiopharmaceutical choice should be matched to clinical indication. Across included meta‐analyses, two consistent patterns emerged: (i) ^18^F‐FDG PET/CT provides clinically useful information in selected scenarios but has limited sensitivity for nodal and peritoneal disease, and (ii) ^68^Ga‐FAPI PET demonstrates higher pooled sensitivity than FDG across key diagnostic indications—particularly for peritoneal involvement—but remains constrained by smaller evidence bases, variable standards, and evolving clinical implementation [12].

### 4.1. FDG‐PET: Established Role, but Indication‐Dependent Limitations

Current international guidance recognizes PET/CT as a useful adjunct rather than a universal first‐line test in gastric cancer work‐up. The NCCN Gastric Cancer guidance (2025) notes that FDG‐PET/CT may be reviewed by multidisciplinary teams “when available,” reflecting both its utility and its nonroutine status in many pathways [19]. Consistent with this pragmatic positioning, pooled results across meta‐analyses show that FDG‐PET/CT is more reliable for confirming advanced disease than for excluding it—especially for nodal disease and peritoneal carcinomatosis [17].

These limitations have biologic and technical explanations. FDG uptake varies with histologic subtype and tumor cellularity, and small‐volume lesions are prone to partial‐volume effects, reducing detection of micrometastatic LN disease and subtle peritoneal spread. This helps explain why FDG frequently shows high specificity but limited sensitivity for LN involvement, whereas performance for peritoneal metastases is often poor [17].

### 4.2. FAPI‐PET: Stronger Diagnostic Signal, Especially for Peritoneum

The most clinically impactful finding in this umbrella review is the consistent diagnostic advantage of FAPI‐based PET compared with FDG in gastric cancer–focused comparative analyses. The largest meta‐analysis dedicated to gastric cancer (Ruan et al. 2023) found higher pooled diagnostic accuracy for ^68^Ga‐FAPI than FDG, with markedly improved pooled sensitivity across primary tumor detection, nodal assessment, peritoneal metastases, and recurrence/restaging scenarios [12]. A second comparative meta‐analysis [14] similarly supports the superiority of ^68^Ga‐FAPI‐04 PET (MRI/CT platforms) over FDG for primary tumor detection, LN metastases, and peritoneal involvement.

This advantage is mechanistically plausible because FAPI tracers target fibroblast activation protein (FAP) on cancer‐associated fibroblasts, often producing high tumor‐to‐background ratios in stromal‐rich tumors and low physiologic uptake in many normal tissues. Contemporary reviews emphasize the expanding clinical footprint of FAPI‐PET across malignancies while also highlighting known pitfalls (uptake in benign inflammatory/fibrotic processes) [20]. These points align well with our indication‐based framework and support prioritizing FAPI‐PET for comprehensive staging/restaging, especially when peritoneal disease is a key concern.

However, an important limitation of FAPI imaging is its nonspecific uptake in benign conditions, including inflammatory processes, fibrosis, and postsurgical or posttherapeutic changes. These false‐positive findings may reduce specificity and should be carefully interpreted in clinical context.

### 4.3. Recurrence Detection: Strong Performance Signals, but Low Certainty Persists

For recurrence detection, FDG‐PET/CT shows moderate sensitivity in gastric cancer–specific meta‐analyses, but heterogeneity is substantial [10]. FAPI‐PET demonstrates very high pooled sensitivity for recurrence in meta‐analyses that include gastrointestinal subgroups, but this evidence is constrained by smaller samples and mixed‐cancer populations [7]. These limitations explain why GRADE certainty for recurrence outcomes remained very low in our synthesis, despite favorable pooled point estimates.

Clinically, this supports a nuanced pathway: FDG‐PET/CT remains useful for recurrence evaluation—particularly when disease is metabolically active—whereas FAPI‐PET may be most valuable for patients with suspected peritoneal or infiltrative recurrence or when conventional imaging is equivocal, recognizing that false positives can arise from benign fibrotic/inflammatory processes [20].

For recurrence detection, FDG‐PET/CT shows moderate sensitivity in gastric cancer–specific meta‐analyses, but heterogeneity is substantial. FAPI‐PET demonstrates very high pooled sensitivity for recurrence in broader meta‐analyses that include gastrointestinal subgroups; however, because these data are not exclusively gastric cancer–specific, the certainty and direct applicability of these findings to gastric cancer remain limited.

### 4.4. Prognosis and Treatment Response: FDG Has Evidence; FAPI Remains Emerging

This umbrella review indicates moderate certainty for FDG‐derived prognostic associations (SUV‐based metrics and survival outcomes), consistent with ongoing literature showing metabolic parameters as prognostic correlates in gastric cancer and related upper GI malignancies. In contrast, no dedicated meta‐analysis currently establishes prognostic utility for FAPI‐PET in gastric cancer, leaving the prognostic role of FAPI imaging unproven at the meta‐analytic level (very low/no evidence in GRADE). This gap represents a high‐value target for future longitudinal and outcomes‐focused studies.

### 4.5. Practical Implications for “Radiopharmaceutical Selection + Clinical Indication”

When translated into practice, the evidence supports a precision imaging approach:

Staging/restaging with high risk of peritoneal disease: prioritize ^68^Ga‐FAPI PET (strong diagnostic signal across meta‐analyses) [12].

Recurrence evaluation: consider FDG‐PET/CT as a widely available option; consider FAPI‐PET in equivocal cases or when peritoneal/infiltrative recurrence is suspected, acknowledging limited certainty and potential false positives [10].

Prognostic stratification/metabolic biology: FDG‐derived quantitative parameters remain the best supported PET biomarkers for survival‐related outcomes in current meta‐analytic evidence [21].

These implications are also consistent with contemporary guideline ecosystems, where FDG‐PET/CT is integrated selectively rather than universally in gastric cancer staging and follow‐up pathways [22]. Preliminary evidence suggests complementary roles of FDG and FAPI, with FDG reflecting tumor metabolism and FAPI reflecting stromal activity; however, robust meta‐analytic validation of dual‐tracer strategies is currently lacking.

### 4.6. Quality, Heterogeneity, and Why Certainty Remains Limited

Despite clear directional findings, the AMSTAR‐2 and GRADE syntheses highlight important constraints. Many diagnostic meta‐analyses show substantial heterogeneity, driven by (i) differences in reference standards (histopathology vs. follow‐up), (ii) patient spectrum (early vs. advanced; histologic mix), (iii) PET acquisition/interpretation variability, and (iv) outcome definitions (patient‐based vs. lesion‐based) [17]. Additionally, for FAPI imaging specifically, newer tracers and protocols are still converging toward standardization; recent professional guidance on FAP PET emphasizes best practices and highlights technical/interpretive considerations—an important step toward improving reproducibility and reducing bias [23]. The quantitative overlap analysis demonstrated slight overlap (CCA = 4.08*%*), suggesting that redundancy across reviews was minimal and that the included meta‐analyses contributed largely independent evidence. Therefore, despite consistent directional signals favoring FAPI for several diagnostic indications, the present conclusions should be interpreted as evidence‐informed comparative judgments rather than as definitive updated pooled effect estimates.

### 4.7. Research Priorities for 2026 and Beyond

To strengthen clinical translation and improve certainty, future studies should prioritize (i) prospective, gastric cancer–specific head‐to‐head comparisons of FDG versus FAPI with standardized reference standards; (ii) explicit reporting of patient‐based and lesion‐based performance by site (LN vs. peritoneum vs. visceral); (iii) standardized thresholds and harmonized reporting of quantitative metrics (SUV/TBR) and timing; and (iv) longitudinal studies linking FAPI uptake patterns to survival, recurrence, and treatment response, enabling true prognostic meta‐analysis. Recent reviews in 2024–2025 emphasize both the promise of FAPI‐PET and the need to address benign uptake, standardization, and clinical integration, supporting these priorities [20].

## 5. Conclusion

The current evidence suggests that radiopharmaceutical selection in gastric cancer should be indication‐driven. FDG‐PET remains a useful and widely available tracer for selected clinical questions, particularly prognostic assessment and some recurrence settings, whereas FAPI‐PET shows a stronger diagnostic signal for staging and restaging, especially for peritoneal disease. However, because part of the available evidence is derived from mixed‐population reviews and newer FAPI studies, further high‐quality, gastric cancer–specific validation is needed before broad guideline integration.

## Author Contributions

Conceptualization: Aiganym Amrenova, Amin Tamadon, and Nadiar M. Mussin; methodology: Aiganym Amrenova, Alma Shukirbekova, Sholpan Akhelova, and Amin Tamadon; literature search and study selection: Aiganym Amrenova, Alma Shukirbekova, and Jamilya Assilbayeva; data extraction and curation: Aiganym Amrenova, Sholpan Akhelova, and Maira Mukazhanova; formal analysis and evidence synthesis: Aiganym Amrenova, Andrey Gurin, Bibigul Kaliyaskarova, and Asset Sarsekeyev; methodological quality and certainty assessment (AMSTAR‐2, GRADE): Aiganym Amrenova and Amin Tamadon; visualization and figures: Aiganym Amrenova and Andrey Gurin; writing—original draft: Aiganym Amrenova and Amin Tamadon; writing—review and editing: Alma Shukirbekova, Nadiar M. Mussin, Sholpan Akhelova, Jamilya Assilbayeva, Andrey Gurin, Maira Mukazhanova, Bibigul Kaliyaskarova, and Asset Sarsekeyev; supervision: Amin Tamadon and Nadiar M. Mussin; project administration: Aiganym Amrenova. All scientific content, interpretations, and final decisions were made by the authors.

## Funding

Institutional support from Astana Medical University covered routine academic and publication‐related expenses; however, this support does not constitute a formal research grant.

## Disclosure

All authors are responsible for the final approval of the manuscript. The authors confirm that they take full responsibility for the content of this manuscript.

## Ethics Statement

The authors have nothing to report.

## Conflicts of Interest

The authors declare no conflicts of interest.

## Data Availability

The raw data supporting the conclusions of this article will be made available by the authors, without undue reservation.
